# Muscle progenitor cells are required for skeletal muscle regeneration and prevention of adipogenesis after limb ischemia

**DOI:** 10.3389/fcvm.2023.1118738

**Published:** 2023-03-02

**Authors:** Hasan Abbas, Lindsey A. Olivere, Michael E. Padgett, Cameron A. Schmidt, Brian F. Gilmore, Timothy J. McCord, Kevin W. Southerland, Joseph M. McClung, Christopher D. Kontos

**Affiliations:** ^1^Department of Pharmacology and Cancer Biology, Duke University Medical Center, Durham, NC, United States; ^2^Duke-NUS Medical School, Singapore, Singapore; ^3^Department of Medicine, Division of Cardiology, Duke University Medical Center, Durham, NC, United States; ^4^Duke University School of Medicine, Durham, NC, United States; ^5^Department of Physiology, Brody School of Medicine, East Carolina University, Greenville, NC, United States; ^6^Brody School of Medicine, East Carolina Diabetes and Obesity Institute, East Carolina University, Greenville, NC, United States; ^7^Department of Surgery, Duke University Medical Center, Durham, NC, United States; ^8^Department of Cell Biology, Duke University School of Medicine, Durham, NC, United States; ^9^Brody School of Medicine, East Carolina Heart Institute, East Carolina University, Greenville, NC, United States

**Keywords:** peripheral artery disease, critical limb threatening ischemia, muscle progenitor cells, skeletal muscle regeneration, fibro/adipogenic progenitor cells, hind limb ischemia, adipogenesis

## Abstract

Skeletal muscle injury in peripheral artery disease (PAD) has been attributed to vascular insufficiency, however evidence has demonstrated that muscle cell responses play a role in determining outcomes in limb ischemia. Here, we demonstrate that genetic ablation of Pax7^+^ muscle progenitor cells (MPCs) in a model of hindlimb ischemia (HLI) inhibited muscle regeneration following ischemic injury, despite a lack of morphological or physiological changes in resting muscle. Compared to control mice (Pax7^WT^), the ischemic limb of Pax7-deficient mice (Pax7^Δ^) was unable to generate significant force 7 or 28 days after HLI. A significant increase in adipose was observed in the ischemic limb 28 days after HLI in Pax7^Δ^ mice, which replaced functional muscle. Adipogenesis in Pax7^Δ^ mice corresponded with a significant increase in PDGFRα^+^ fibro/adipogenic progenitors (FAPs). Inhibition of FAPs with batimastat decreased muscle adipose but increased fibrosis. *In vitro*, Pax7^Δ^ MPCs failed to form myotubes but displayed increased adipogenesis. Skeletal muscle from patients with critical limb threatening ischemia displayed increased adipose in more ischemic regions of muscle, which corresponded with fewer satellite cells. Collectively, these data demonstrate that Pax7^+^ MPCs are required for muscle regeneration after ischemia and suggest that muscle regeneration may be an important therapeutic target in PAD.

## 1. Introduction

Peripheral artery disease (PAD) is caused by atherosclerosis of the peripheral arteries, most commonly the legs. PAD affects over 200 million individuals globally, and it is a major contributor to disease burden in both developing and developed countries (1, 2). Current treatment options are limited to surgical and percutaneous revascularization approaches (3), both of which have minimal impact on long-term morbidity and mortality (4, 5). The clinical course of PAD ranges from the milder manifestation of intermittent claudication (IC), resulting in pain with ambulation that resolves with rest, to the more severe critical limb threatening ischemia (CLTI), characterized by pain at rest, either with or without tissue necrosis (3). Although CLTI affects only 10–15% of patients with PAD, it results in a substantial burden on the health care system, as these patients often progress to limb amputation and have significantly greater morbidity and mortality (6, 7). Although therapeutic approaches to PAD primarily target revascularization and tissue perfusion, it has been observed that patients with similar degrees of atherosclerotic vascular occlusion often present with markedly different severity of disease (8, 9), suggesting that blood flow alone may not determine clinical outcomes.

Recent evidence from our group and others supports the idea that skeletal muscle responses to tissue ischemia, and not solely the vascular supply, play an important role in determining the muscle response to limb ischemia (9–12). In mice subjected to hind limb ischemia (HLI), a model of PAD, the genetic background strongly influences outcomes. For example, C57BL/6 mice display not only robust angiogenesis but also a muscle regenerative response that typically leads to full recovery from HLI. In stark contrast, HLI in BALB/c mice typically results in muscle degeneration and auto-amputation, and even muscle that does survive fails to recover function, i.e., force generation (9). Although this genetic difference was previously attributed to differences in collateral vessel density (13–16), muscle progenitor cells (MPCs) isolated from these strains of mice display markedly different responses to experimental ischemia *in vitro*, independent of blood supply. This finding is consistent with the differential responses observed *in vivo* and it suggests muscle cell-specific determinants of the response to ischemia. Although the mechanisms by which skeletal muscle responds to ischemia remain poorly understood, a genetic variant in at least one gene, *Bag3*, has been linked to this differential ischemic response in mice (12). However, it is not known whether these effects are at the level of mature muscle cells or MPCs.

MPCs, commonly known as satellite cells, lie between the basal lamina and plasma membrane of skeletal muscle cells and are critical regulators of postnatal myofiber regeneration (17, 18). MPCs are defined by expression of the Pax3 homolog Pax7, and they serve as a unipotent stem cell population for myogenesis following injury (19). However, these cells have a limited capacity for self-renewal, and repeated replication cycles may result in depletion of the satellite cell pool (20). The development of genetically modified mouse models to ablate MPCs has allowed investigation of the role of Pax7^+^ MPCs in various disease states. In particular, mice inducibly expressing *Diphtheria* toxin A (DTA) only in Pax7^+^ cells have been used to demonstrate a requirement for these MPCs in muscle regeneration in a variety of conditions. Most of these studies have been performed using cytotoxic injury models, such as cardiotoxin, freeze injury, or BaCl_2_ injury (21). However, it is known that different modes of injury have unique characteristics. For example, glycerol injury results in a more adipogenic phenotype compared to other modes of injury (22). Although we and others have characterized the skeletal muscle response to ischemia (9, 10, 12, 23, 24), the role of MPCs in this process remains unexplored.

In addition to MPCs, the discovery of a novel subpopulation of fibro/adipogenic progenitors (FAPs) in mature skeletal muscle (25) has led to considerable focus on the role of these cells in pathological skeletal muscle conditions. Histologically and functionally, these cells can be identified in skeletal muscle by their expression of platelet-derived growth factor receptor alpha (PDGFRα) and signaling by this receptor in pathophysiology (26–28). FAPs isolated from skeletal muscle were able to cause white fat infiltration in diseased but not in healthy muscle because myofibers have a significant inhibitory effect on the differentiation of FAPs (29). This observation suggests an environmental contribution to FAP cell fate. FAP expansion has also been shown to regulate the MPC pool during muscle regeneration in addition to playing a critical role in skeletal muscle homeostasis (27).

Here, we used genetically modified mouse models to explore the role of Pax7^+^ MPCs in the skeletal muscle response to hind limb ischemia. We demonstrate a near complete absence of skeletal muscle regeneration after HLI in mice following ablation of Pax7^+^ satellite cells. Furthermore, ischemic, Pax7-deficient muscle displayed a dramatic increase in adipogenesis that was driven at least in part by FAPs. Consistent with these findings in mice, decreased MPC numbers and increased adipogenesis were observed in more ischemic regions of skeletal muscle of CLTI patients. These findings demonstrate the requirement for Pax7^+^ MPCs in ischemic skeletal muscle regeneration, and they provide important new insights into the pathogenesis of PAD.

## 2. Materials and methods

### 2.1. Mouse lines and tamoxifen treatment

For satellite cell genetic ablation experiments, Pax7-Cre^ERT2^ mice (Jackson Labs Stock: 017763, B6.Cg-*Pax7^tm1(cre/ERT2)Gaka^*/J) were crossed to ROSA26^DTA^ mice (Jackson Labs Stock: 009669, C.129P2(B6)-*Gt(ROSA)26Sor^tm1(DTA)Lky^*/J). Both lines of mice had been backcrossed to C57BL/6 J mice for at least 8 generations at the time of these studies. Mice were given sterile-filtered tamoxifen (Sigma T5648) or corn oil at 75 mg/kg body weight *via* an intraperitoneal route for 5 days. Following this initial treatment, mice were given tamoxifen (Envigo Teklad Tamoxifen Diet TD.130855) or a control-matched diet (Envigo Teklad global 16% protein diet 2016S) to continue treatment at a lower dose of 50 mg/kg body weight. All mice were used at 8–12 weeks of age unless stated otherwise.

### 2.2. Hindlimb ischemia surgery and perfusion imaging

Hindlimb Ischemia surgery was performed as described previously (10, 30). Briefly, mice were anaesthetized on a heated pad with inhaled isoflurane (1–3%) in oxygen (1.5 L/min). Prior to surgery, the mice were scanned with a Laser Doppler Perfusion Imager (LDPI, Moor Instruments United States) to quantify baseline perfusion in the hindlimbs. Using sterile surgical instruments (sterilized by autoclaving), a 1-cm incision was made just below the inguinal ligament. Subcutaneous fat was removed and the femoral artery was separated from the neurovascular bundle, taking care not to perforate the femoral vein. A 7–0 silk non-absorbable suture (Sharpoint) was used to ligate the femoral artery above the bifurcation of the lateral circumflex femoral artery, and a ligature was also made below the superficial caudal epigastric artery but above the bifurcation of the popliteal artery. The wound was then closed using an absorbable Vicryl 5–0 suture (Ethicon). A post-operative LDPI scan was then performed to verify complete occlusion of the artery. Mice were provided appropriate pain relief and monitored after surgery to ensure animal welfare.

### 2.3. Tissue collection and muscle processing

Mice were deeply anesthetized with inhaled isoflurane as described above, and the tibialis anterior (TA) and extensor digitorum longus (EDL) muscles were isolated and frozen on liquid nitrogen in Optimal Cutting Temperature (OCT) medium, while the gastrocnemius muscles were flash frozen in liquid nitrogen and stored at −80°C for later tissue analysis. Mice were euthanized by exsanguination or bilateral thoracotomy while still under anesthesia. Tissue sections (8 μm or 30 μm) were cut on a Leica 3150S cryostat at −21° to −25°C and stored on Superfrost Slides.

### 2.4. Immunofluorescence microscopy and image analysis

Tissue sections were fixed in 4% paraformaldehyde (PFA) followed by permeabilization in 0.2% Triton X-100 in Phosphate-Buffered Saline (PBS). After washes in PBS, slides were blocked with 5% normal goat serum (NGS) in PBS for 1 h. Dilutions of antibodies used for immunostaining are listed in the Table 1. For Pax7 immunofluorescence staining, a goat anti-mouse IgG blocking antibody (Jackson Immunoresearch 115–007-003) was used in blocking buffer, and an antigen retrieval step was performed by heating slides in a Cuisinart CPC-6001000-watt pressure cooker at high setting in 10 mM sodium citrate, 0.05% Tween 20, pH 6.0, and allowed to return to room temperature over 20 min prior to incubation with the primary antibody. Slides were incubated overnight at 4°C with antibodies of interest at the dilutions listed in Table 1. The following day, sections were washes 3 times in PBS, followed by incubation at room temperature for 1 h with appropriate Alexa Fluor-conjugated secondary antibody. Slides were then washed 3 times with PBS or PBST followed by a 5-min incubation with a nuclear stain (DAPI or Hoechst) as indicated. After a final PBS rinse, slides were mounted in either Vectashield Antifade Mounting Medium (H1000) or Prolong Gold Antifade Mountant (Invitrogen P36962) and allowed to cure overnight. Brightfield microscopy and epifluorescence microscopy were both performed on a Zeiss Upright AxioImager, while all confocal microscopy was performed on the Zeiss 780 or Zeiss 880 Inverted Confocal Microscope. All image analysis was performed in Zeiss Zen software, IMARIS, or ImageJ with identical thresholds and blinding performed for all signal quantification.

**Table 1:** Commercially available antibodies used for immunofluorescence microscopy.

Antibody	Manufacturer	Catalog Number	Dilution	Species
Pax7	DSHB	Pax7	1:50	Mouse IgG1
Dystrophin	Thermo	RB-9024	1:100	Rabbit
Type IIa fibers	DSHB	SC-71	1:100	Mouse IgG1
Type I fibers	DSHB	BA-F8	1:100	Mouse IgG2b
Type IIb fibers	DSHB	BF-F3	1:100	Mouse IgM
Embryonic myosin heavy chain	DSHB	F1.652	1:50	Mouse IgG1
CD31	BioRad	MCA2388	1:200	Rat
Dystrophin	Abcam	ab3149 (discontinued)	1:100	Mouse IgG1
Perilipin	Cell Signaling Technologies	9349S	1:200	Rabbit
PDGFRα	Cell Signaling Technologies	3174S	1:800	Rabbit
Myogenin	DSHB	F5D	1:50	Mouse IgG1
Myosin heavy chain	DSHB	A4.1025	1:200	Mouse IgG2a

### 2.5. Hematoxylin and eosin staining

Sections were brought to room temperature, then fixed for 10 min in 10% Neutral Buffered Formalin (NBF). The slides were washed with distilled water followed by staining of nuclei with Meyer’s hematoxylin for 4 min. Slides were rinsed under running tap water for 10 min and then differentiated with 0.3% acid-alcohol. An additional rinse with tap water and Scott’s tap water substitute was used to further enhance coloration of the nuclei. Samples were then briefly incubated in 90% ethanol (EtOH) and then stained with alcoholic eosin for 30 s. Slides were then dehydrated in 100% EtOH followed by 2 rinses in xylene or xylene substitute for 2 min each before mounting with Cytoseal resin-based mounting medium. The slides were then allowed to cure overnight prior to imaging.

### 2.6. Muscle contractile measurements

Contractile muscle force was measured as described previously (31). Briefly, single EDL muscles were isolated and ligated with a 5–0 silk suture at each tendon and maintained in a physiological saline solution (pH 7.6) containing 119 mM NaCl, 5 mM KCl, 1 mM MgSO_4_, 5 mM NaHCO_3_, 1.25 mM CaCl_2_, 1 mM KH_2_PO_4_, 10 mM HEPES, and 10 mM dextrose at 30°C under aeration with 95% O_2_/5% CO_2_ throughout the experiment. Muscles were mounted in a bath within the force transducer (Aurora 300B-LR) operated in isometric mode. A 5-min equilibration was performed, during which single twitches were elicited every 30 s with 0.5 msec electrical pulses. Isometric tension was evaluated by 250 msec trains of pulses delivered at 10, 20, 40, 60, 80, 100 and 120 Hz. After the experimental protocol, muscle length was determined with a digital caliper and muscle mass was measured after removing liquid. The cross-sectional area for each muscle was measured, and muscle density was determined as the muscle mass (g) divided by the product of its length (L_o_, mm) and cross-sectional area (mm^2^), expressed in g/mm^3^. Muscle output was then expressed as isometric tension (N/cm^2^) determined by dividing the developed tension (N) by the muscle cross-sectional area. In the case of atrophied muscle, absolute tension was used as the measure of force because the cross-sectional muscle area is no longer a reliable measure due to change in muscle density.

### 2.7. Oil red O staining

Tissue sections were fixed in 10% NBF for 4 min and briefly washed under running tap water for 1 to 10 min. After rinsing with 60% isopropanol, samples were stained with freshly prepared and filtered oil red O working solution (Oil Red O powder [Sigma] in 60% isopropanol) for 15 min, then rinsed again with 60% isopropanol. The samples were then lightly stained with Meyer’s hematoxylin and rinsed with distilled water. Slides were mounted in aqueous glycerine jelly and imaged within 2 h.

### 2.8. BODIPY staining

Frozen sections were fixed with 4% PFA in PBS for 10 min followed by 2 washes with PBST for 5 min each. The slides were then incubated for 60 min with 1 μg/ml BODIPY 493/503 (Invitrogen D3922) in PBST. Following incubation, the slides were washed twice with PBST for 5 min each, then twice with PBS for 5 min each. Slides were finally mounted with Prolong Diamond Antifade Mountant with DAPI (Invitrogen P36962) and imaged immediately.

### 2.9. MicroCT/DiceCT

EDLs were isolated from hindlimbs of mice and fixed immediately in 10% NBF solution overnight. Muscles were then stained using the diceCT protocol, as described previously (32). Briefly, muscles were incubated in Lugol’s Iodine for 2 nights. The muscles were then scanned in a fixed container at low power in a Nikon XTH 225 ST microCT scanner at a 10-μm or 14-μm resolution. Images were then reconstructed using Nikon automated reconstruction software and analyzed using Avizo to delineate soft tissue densities in false colors.

### 2.10. Batimastat treatment

Pax7-Cre^ERT2^; ROSA26^DTA^ mice were all given tamoxifen (75 mg/kg body weight) *via* i.p. injection for 5 days prior to surgery. 1 day prior to surgery, half the mice were given batimastat (30 mg/kg body weight) as a 3 mg/ml suspension in sterile-filtered PBS with 0.01% Tween-80 *via* i.p. injection, and the other half were injected with vehicle only. Batimastat injections were given daily until muscle was isolated. Following surgery, the mice were switched to a tamoxifen diet and the muscle was harvested 7 days post-operatively.

### 2.11. Fast Green/Sirius Red staining

Samples were placed in 0.04% Fast Green (Sigma) for 15 min then washed with distilled water. Sections were then incubated in 0.1% Fast Green and 0.04% Sirius Red (Sigma) in saturated picric acid for 30 min. Samples were dehydrated through serial 70, 90, and 100% Ethanol washes and cleared in xylene for 2 min before mounting using Cytoseal mounting medium. Positive and negative controls were run simultaneously to validate the specificity of this assay for collagen.

### 2.12. Myoblast isolation

Hindlimb muscles from mice were dissected, rinsed briefly in sterile PBS, and placed in a 10-cm dish containing DMEM +1% penicillin/streptomycin (pen/strep). Thereafter, all steps were performed in a biosafety cabinet under sterile conditions. Muscles were cleaned of excess connective tissue and tendons and transferred to a new 10-cm dish containing 5 mL DMEM +1% pen/strep. The muscle was minced with razor blades for >10 min then transferred to a 50-ml centrifuge tube using a wide-bore pipet. Samples were centrifuged in a tabletop centrifuge for 2 min at 800× *g*. The medium was aspirated, cells were resuspended in 18 mL DMEM + pen/strep, and 2 ml pronase (1% solution) was added and the mixture was digested for 1 h at 37°C on a Nutator. The cells were then centrifuged for 3 min at 800 × *g* and the medium was aspirated. Muscle was then suspended in 10 mL DMEM +10% FBS + pen/strep and triturated 20 times to loosen cells. The supernatant was filtered through a Steriflip 100-μm vacuum filter and washed with 5 ml DMEM with 10% FBS + pen/strep. The cells were then centrifuged 5 min at 1,000× *g* and resuspended in 10 mL of growth medium (Ham’s F10 with 20% FBS + pen/strep) and plated on collagen-coated plates.

### 2.13. *In vitro* myogenic and adipogenic differentiation

Differentiation of isolated myoblasts was stimulated by plating the cells on entactin-collagen-laminin-coated plates in differentiation medium (DMEM supplemented with 2% horse serum, 1% pen/strep, 0.2% amphotericin B, and 0.01% human insulin/transferrin/selenium). Effects of hypoxia were determined by placing cells in a hypoxia chamber (Billups-Rothenberg) at 0% O_2_ (95% N_2_, 5% CO_2_). Control cells were maintained in normoxia (21% O_2_, 5% CO_2_). Medium was changed daily to ensure cell viability. Adipogenic differentiation was induced by incubating cells for 48 h in medium containing 10% FBS, 0.5 mM isobuylmethylxanthine, 125 nM indomethacin, 1 μM dexamethosone, 850 nM insulin, 1 nM T3 with or without 1 μM rosiglitazone. After 48 h, cells were switched to medium containing 10% FBS, 850 nM insulin, 1 nM T3, and 1 μM rosiglitazone. Cells were placed in either normoxic or hypoxic conditions as described above, and medium was changed every other day to ensure cell viability.

### 2.14. Human skeletal muscle acquisition

Critical limb threatening ischemia (CLTI) patients undergoing above-or below-knee amputations were consented according to an Institutional Review Board (IRB)-approved protocol to donate skeletal muscle tissue from the amputated limb. Muscle samples were collected under sterile conditions in the operating room from both the proximal and distal ends of the gastrocnemius muscle and oriented cross-sectionally in OCT and frozen on liquid nitrogen. The samples were then sectioned in a cryostat and stored at −80°C for subsequent analysis.

### 2.15. Statistical analysis

For each of the analyses, a script was used to blind the reviewer to either the images or the animal treatments to ensure no bias in the analysis. For a comparison of 2 groups, a two-way Student’s *t*-test was performed in GraphPad Prism, and statistical significance was established at *p* < 0.05. For multiple group comparisons, an ANOVA was first performed to determine whether an effect was present, followed by a *t*-test for multiple groups with a correction for multiple group testing in GraphPad Prism. Significance was once again established by a corrected *p* value <0.05.

## 3. Results

To study the role of Pax7^+^ MPCs in a mouse model of PAD, we crossed Pax7-Cre^ERT2^ mice to ROSA26^DTA^ mice. To ablate satellite cells, we injected tamoxifen (Pax7^Δ^) or corn oil as a control (Pax7^WT^) for 5 days, followed by femoral artery ligation to induce HLI. Perfusion imaging demonstrated an identical injury and similar perfusion of the ischemic hind limb up to 28 days after HLI surgery in both groups (Supplementary Figure S1C). To maintain MPC ablation, mice were fed a diet supplemented with either corn oil or tamoxifen. To validate the model, muscle sections from the non-ischemic tibialis anterior (TA) muscle were stained for the satellite cell marker Pax7, and in the tamoxifen-treated mice there was a complete absence of satellite cells (Supplementary Figures S1A,B), demonstrating successful ablation of all satellite cells within skeletal muscle.

### 3.1. Satellite cell ablation does not alter resting muscle morphology or physiology

**Figure 1:**
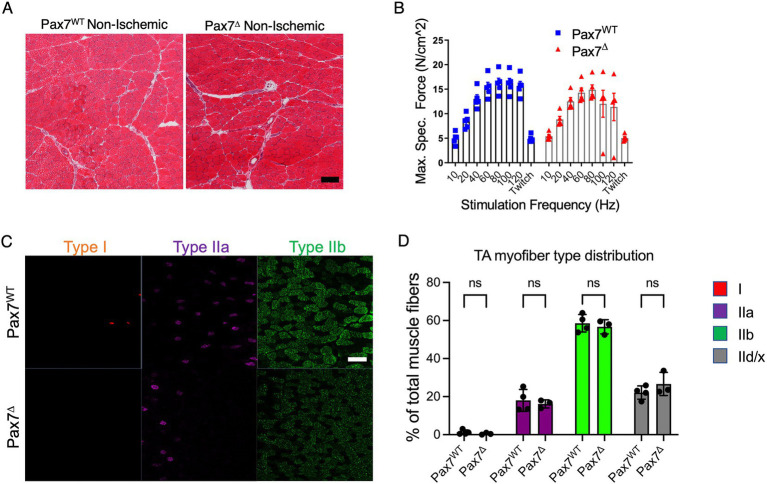
Pax7^+^ MPC ablation does not alter resting muscle morphology 1 week after ischemia. **(A)** H&E stains of skeletal muscle from mice with ablated satellite cells are not distinguishable from those with intact satellite cells (*n* = 3–4 per group). **(B)** EDL muscles from mice (*n* = 5 per group) were isolated, and their ability to generate force was measured on a force transducer. Ablation of satellite cells did not impair the ability of resting skeletal muscle to generate force. Data shown are means +/− SEM. **(C)** Representative immunostains of type I, IIa, and IIb myofibers in non-ischemic limbs of Pax7^WT^ and Pax7^Δ^ mice. **(D)** Quantification of relative percentages of each myofiber type in non-ischemic muscle of Pax7^WT^ and Pax7^Δ^ mice. Pax7^+^ MPC ablation did not alter non-ischemic resting muscle fiber type distribution 1 week after ischemia (*n* = 3–4 per group). Type IId/x myofibers were quantified by lack of staining for the other three markers. All data shown are means +/− SEM; p = ns for all comparisons. Scale bar = 100 μm.

To examine whether satellite cell ablation resulted in changes in resting muscle, we isolated the TA muscle from the contralateral, non-ischemic limbs of Pax7^Δ^ and Pax7^WT^ mice after HLI and compared the skeletal muscle histologically by H&E staining. Muscle morphology and architecture appeared similar in Pax7^Δ^ and Pax7^WT^ mice (Figure 1A), although total cross-sectional area of the TA muscle was significantly reduced in Pax7^Δ^ mice (4.48 ± 0.24 mm^2^ vs. 5.49 ± 0.07 mm^2^, *p* = 0.0043), possibly due to greater muscle hypertrophy in Pax7^WT^ mice after disuse of the ischemic limb. Importantly, however, *ex vivo* force generation of extensor digitorum longus (EDL) muscle did not differ between Pax7^Δ^ and Pax7^WT^ mice (Figure 1B), demonstrating that absence of satellite cells does not alter resting muscle physiology. Lastly, we examined whether deletion of the endogenous skeletal muscle progenitor cell pool affects muscle fiber type distribution. Staining and quantification of both slow-twitch type 1 fibers, which are highly oxidative, and more glycolytic type IIa, IIb, and IId/x fibers (which are more abundant in TA muscle) demonstrated no differences between the groups, demonstrating that loss of Pax7^+^ MPCs does not cause a shift in myofiber metabolism at rest (Figures 1C,D).

### 3.2. Satellite cell ablation in ischemic muscle results in complete absence of regeneration 1 week after ischemia

**Figure 2:**
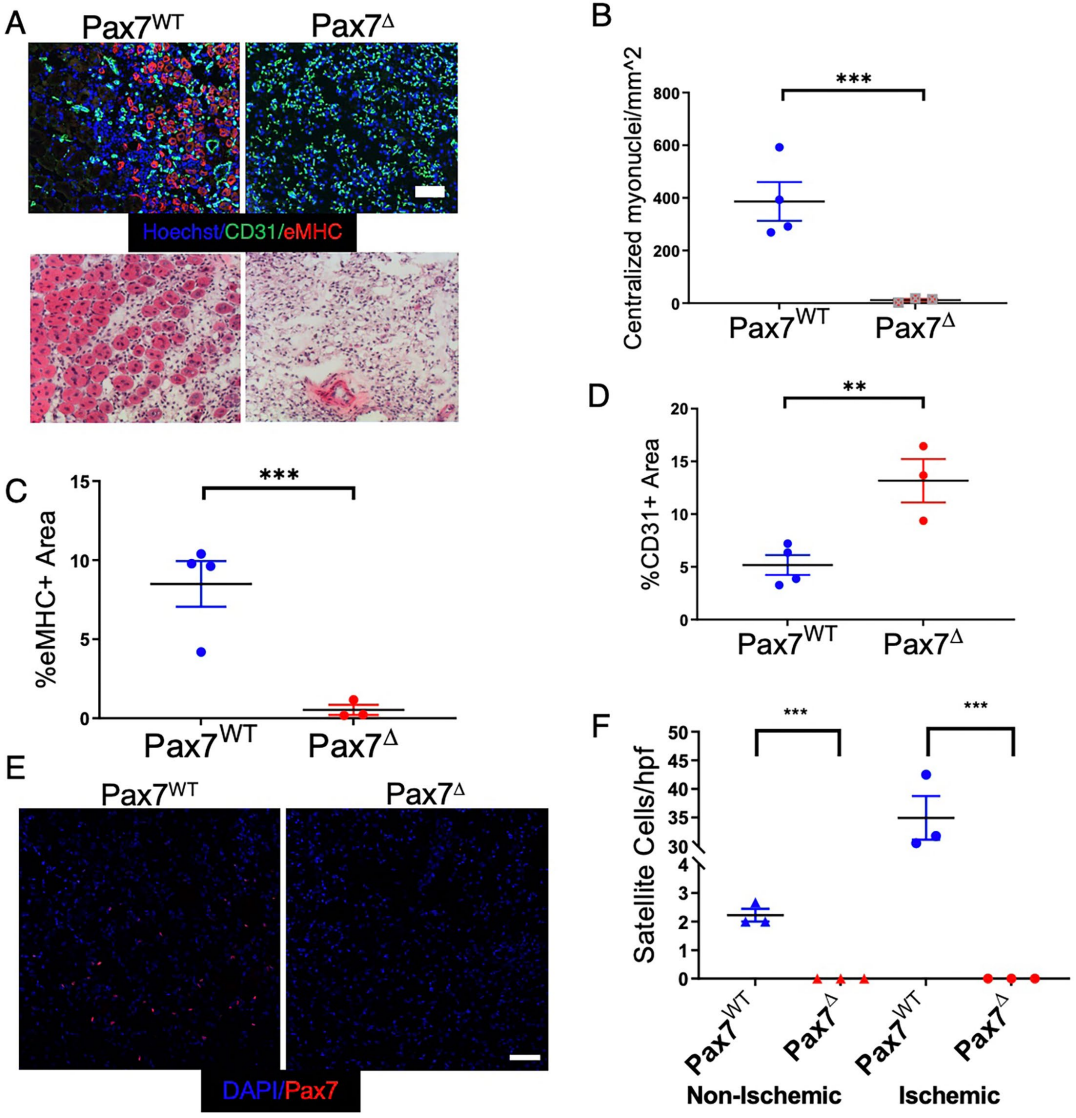
Ablation of Pax7^+^ MPCs in mice results in a complete lack of a muscle regenerative response 1 week after HLI. **(A)** Muscle regeneration was examined by staining for embryonic myosin heavy chain (eMHC, red) and endothelial cells (CD31, green), *top*, and for centralized myonuclei by H&E, *bottom*. **(B–D)** Quantification of centralized myonuclei **(B)** and eMHC **(C)** demonstrates a complete lack of regenerative response to ischemia. Quantification of CD31 area **(D)** demonstrates an increase in endothelial area relative to muscle area in Pax7^Δ^ mice (*n* = 3–4 per group). **(E,F)** 1 week after HLI surgery, there was a significant increase in the number of Pax7^+^ cells per high power field in the ischemic TA of Pax7^WT^ mice but not in muscle of Pax7^Δ^ mice. Compared to resting muscle, there was a 10-15-fold increase in the number of Pax7^+^ cells in injured Pax7^WT^ muscle, consistent with activation of satellite cells following injury (*n* = 3 per group). Scale bar = 100 μm. All data shown are means +/− SEM. **p < 0.01; ***p < 0.001, by 2-sided *t*-test.

To determine the effect of MPC ablation after ischemia, Pax7^Δ^ and Pax7^WT^ mice were subjected to unilateral HLI and examined 7 days later. Following ablation of satellite cells, markers of skeletal muscle regeneration (embryonic myosin heavy chain expression and centralized myonuclei) were absent in the ischemic limb of Pax7^Δ^ mice (Figures 2A–C). To exclude the possibility that genetic ablation of MPCs with DTA had a non-specific effect on the vasculature, muscle sections were stained for the endothelial cell marker PECAM (CD31). Not only was the endothelium intact, but the total endothelial area relative to the muscle area was in fact increased in Pax7^Δ^ mice (Figure 2D), suggesting a possible vascular compensation for the muscle loss. Satellite cell activation and proliferation normally occur after muscle injury in general and are observed after limb ischemia as well. 1 week after HLI, Pax7^WT^ mice displayed a significant increase in the number of Pax7^+^ cells in ischemic TA muscle in contrast to the contralateral, non-ischemic limb, confirming normal satellite cell activation in this model (Figures 2E,F). As expected, this activation was absent in Pax7^Δ^ mice lacking satellite cells, consistent with their inability to regenerate muscle following ischemic injury (Figures 2E,F).

### 3.3. Chronic satellite cell ablation in ischemic muscle results in complete absence of regeneration 1 month after ischemia

**Figure 3:**
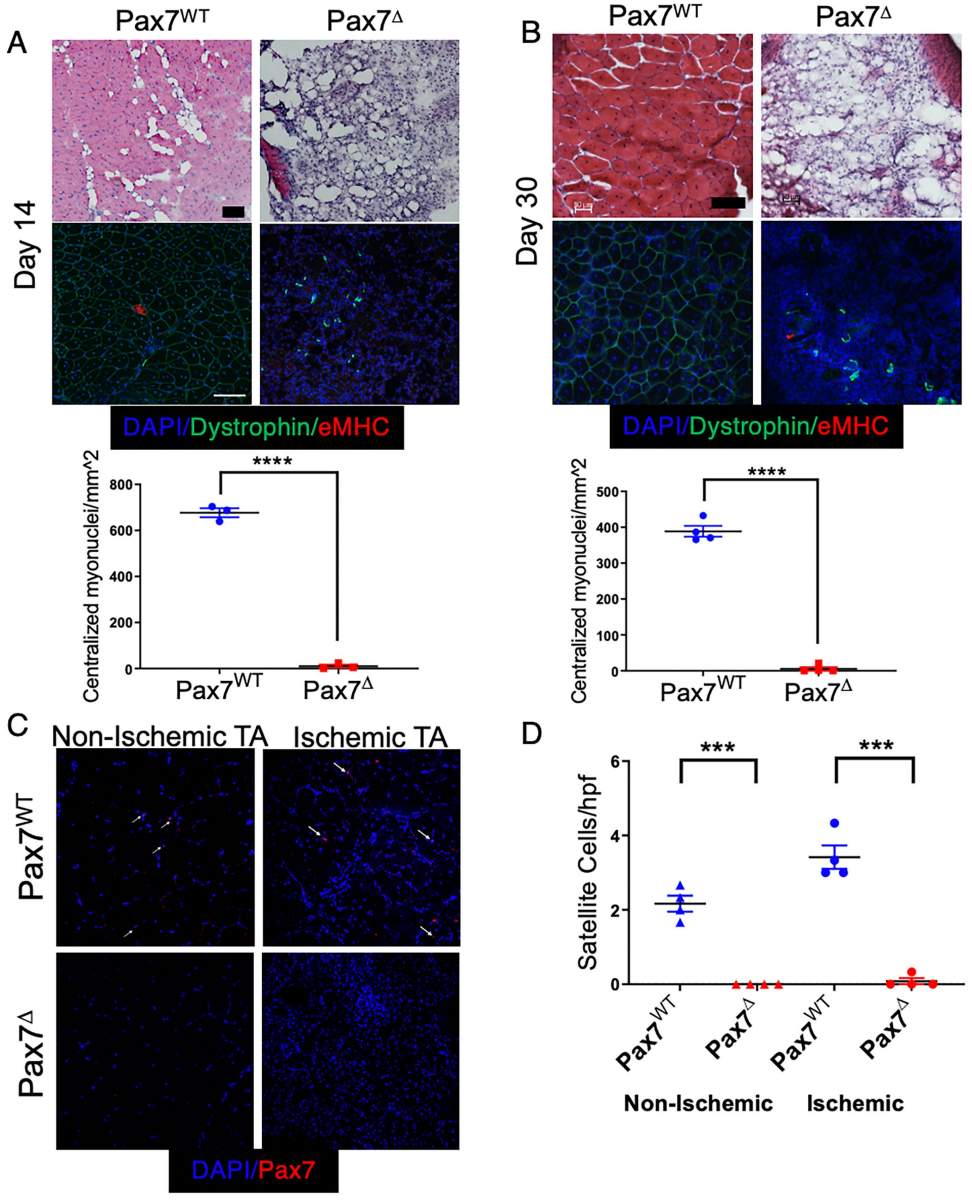
Sustained deletion of satellite cells results in long-term prevention of muscle regeneration after HLI. **(A,B)** H&E staining and quantification of regenerating fibers, demonstrated by myofibers with centralized myonuclei, of the ischemic TA muscle at 14 days **(A)** and 30 days **(B)** after ischemia demonstrated a complete lack of regeneration (*n* = 3–4 per group). **(C,D)** 30 days after HLI Pax7^+^ cells per high power field were significantly increased (2-fold) in the ischemic relative to the non-ischemic TA muscle of Pax7^WT^ mice, although their numbers were diminished compared to 7 days post-HLI. Pax7^+^ cells were persistently absent in Pax7^Δ^ mice (*n* = 4 per group). Scale bar = 100 μm. All data shown are means +/− SEM. ***p < 0.001, ****p < 0.0001 by 2-sided *t*-test.

To investigate the effects of satellite cell ablation on long-term muscle recovery from ischemia, Pax7^Δ^ and Pax7^WT^ mice were subjected to HLI and followed for 14 and 30 days after surgery. Consistent with responses observed in parental C57BL/6 mice, ischemic Pax7^WT^ mice displayed improved muscle architecture at day 14 post-HLI. Expression of eMHC had resolved by this time point, although there were still centralized myonuclei, and an inflammatory infiltrate was still present in the interstitial spaces between muscle fibers (Figure 3A). These features were further improved by day 30, with near compete resolution of inflammation (Figure 3B). In contrast, Pax7^Δ^ mice displayed a persistent absence of muscle regeneration with an accompanying increase in cellularity characteristic of ongoing inflammation (Figures 3A,B). Strikingly, muscle of late stage ischemic Pax7^Δ^ mice displayed a dramatic increase in adipose observed both histologically and by microCT (Figure 3A,B; Supplementary Figure S2), which was also evidenced grossly by the inability of whole muscle tissue to sink in aqueous solution (Supplementary Figure S2A). Whereas distinct, individual myofibers were visualized by microCT in control muscle (Supplementary Figure S2B), EDL muscle from Pax7^Δ^ mice was markedly atrophied and displayed significant soft tissue adipogenic changes (Supplementary Figure S2B). These findings suggested that the chronic absence of satellite cells after ischemic injury resulted not only in a loss of muscle regeneration but also a shift in the cellular makeup of injured muscle. Persistent satellite cell ablation in Pax7^Δ^ mice 30 days after HLI was verified by Pax7 immunostaining (Figures 3C,D). In the non-ischemic limb of Pax7^WT^ mice, satellite cell numbers were similar to the day 7 timepoint, whereas satellite cell number diminished significantly in the ischemic limb by day 30 (~4/hpf compared to ~35/hpf on day 7 post-HLI) and was only slightly higher than in the non-ischemic limb at this stage (Figures 3C,D).

### 3.4. Long-term satellite cell ablation in ischemic muscle results in impaired force generation

**Figure 4:**
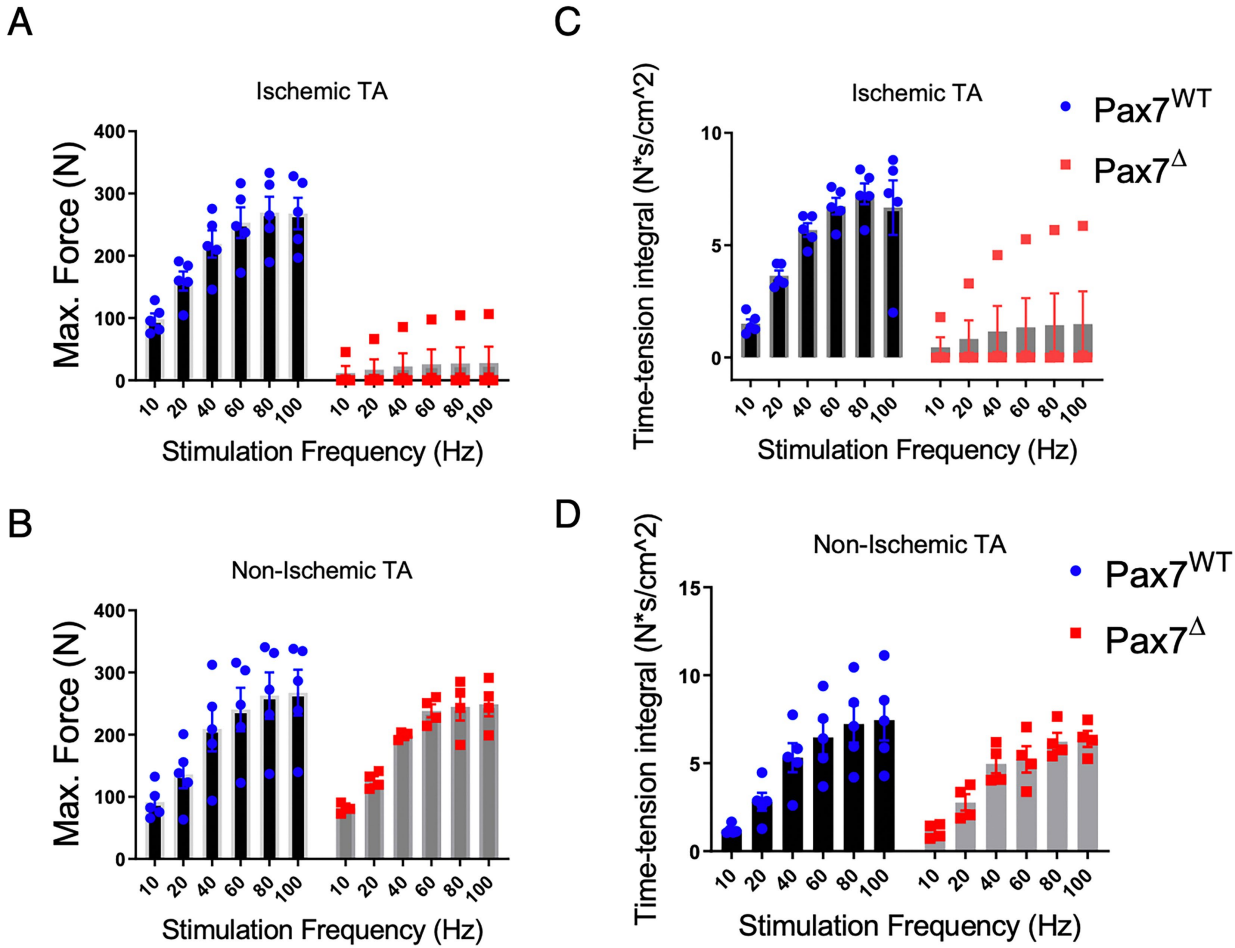
Pax7^+^ MPC ablation impairs force generation 30 days after HLI. **(A)** The maximum force generated by the ischemic EDL muscle was significantly lower (*p* < 0.0001 by 2-way ANOVA) in Pax7^Δ^ mice. **(B)** Maximum force was unchanged in the non-ischemic limb of Pax7^Δ^ mice. **(C,D)** The time-tension integral, a measure of work done in a single contraction, of muscle 30 days after HLI mirrored the maximum force data in both ischemic **(C)** and non-ischemic TA muscle **(D)** (*n* = 4–5 per group).

Because long-term satellite cell ablation resulted in markedly abnormal muscle tissue morphology, we tested *ex vivo* muscle force generation to determine the functional effects of this injury. Force generation in Pax7^Δ^ and Pax7^WT^ mice correlated with histological findings, as there was a significant impairment in both maximal force generation and the time-tension force integral in EDL muscle of Pax7^Δ^ mice compared to that of Pax7^WT^ mice 30 days after ischemia (Figures 4A,B). In stark contrast, force generation in the non-ischemic EDL mirrored that observed on day 7 post-HLI (Figures 4C,D), confirming that resting skeletal muscle is unaffected by satellite cell ablation even after 30 days.

### 3.5. Ablation of Pax7^+^ MPCs in mice results in marked fat infiltration of skeletal muscle following ischemia

**Figure 5:**
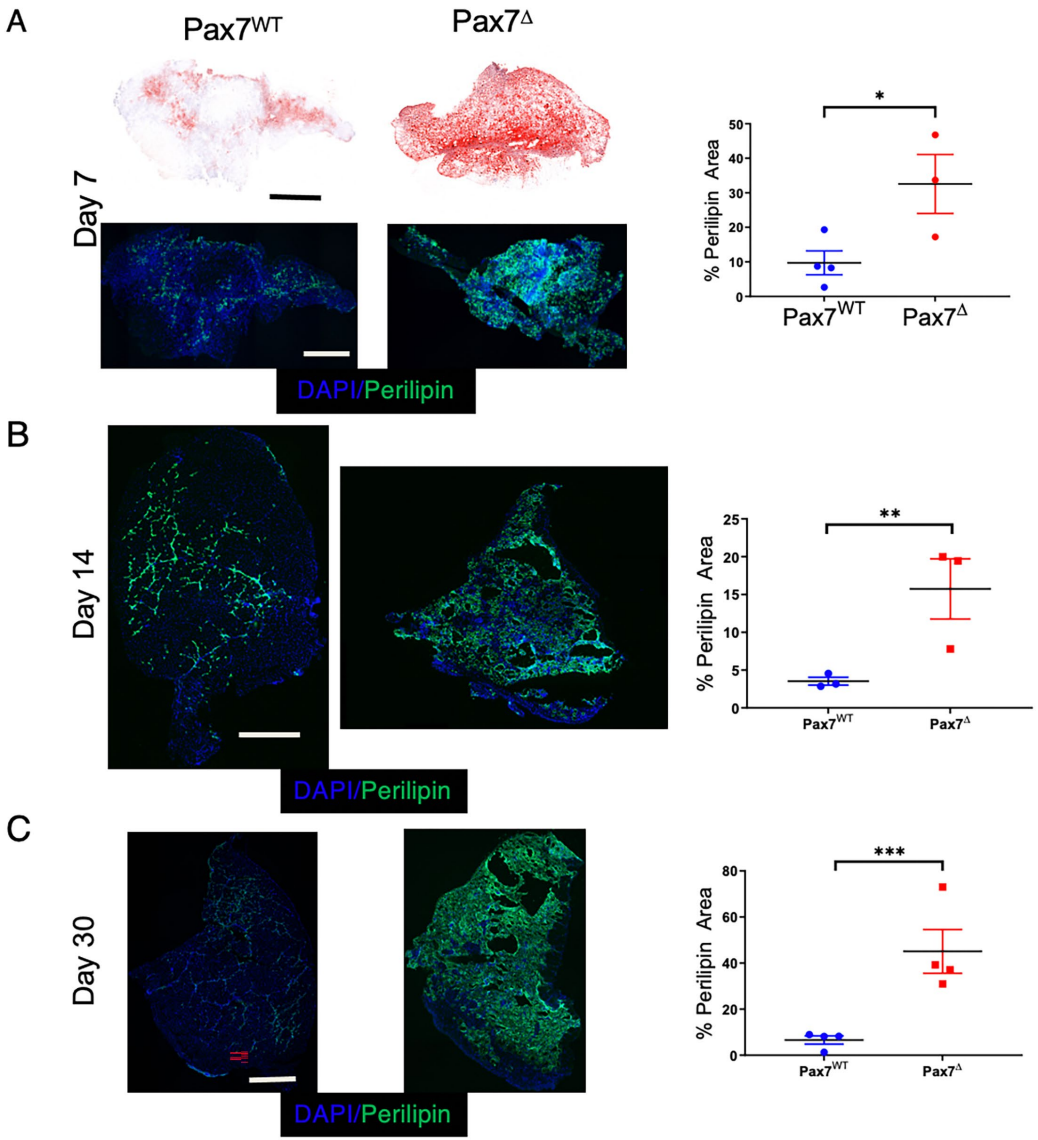
Ablation of Pax7^+^ MPCs in mice results in marked fat infiltration within skeletal muscle following ischemia. **(A)** Oil red O (*top*) and Perilipin (*bottom*) staining of the ischemic TA muscle demonstrated significantly increased lipid staining in Pax7^Δ^ mice compared to Pax7^WT^ 7 days after HLI surgery (*n* = 3–4 per group). **(B,C)** Perilipin staining and quantification of adipose in the ischemic TA muscle 14 days **(B)** and 30 days **(C)** after HLI surgery demonstrated increased lipid staining in Pax7^Δ^ mice compared to Pax7^WT^ (*n* = 3–4 per group). Scale bars = 1 mm. All data shown are means +/-SEM. * represents *p* < 0.05, **p < 0.01; ***p < 0.001 by 2-sided *t*-test.

A key feature of muscle injury is that different modes of injury can result in varying regenerative responses. For example, unlike cardiotoxin-mediated injury, glycerol injection induces a more adipogenic change to the muscle (22). In contrast, the mdx mouse, a genetic model of muscular dystrophy, fails to accurately recapitulate many of the adipogenic changes observed in patients with muscular dystrophy. The lipid deposition seen in Pax7^Δ^ mice 30 days after HLI is reminiscent not only of that of patients with muscular dystrophy but also of patients with CLTI (33). To investigate the adipogenic changes that occur in skeletal muscle following ischemic injury, we used two different complementary lipid stains, oil red O and BODIPY 493/503, to examine fatty changes 7 days after HLI. Oil red O staining showed a small amount of fat deposition in the control Pax7^WT^ TA muscle, which was significantly increased in Pax7^Δ^ muscle (Figure 5A), and these findings were mirrored by the BODIPY staining (data not shown). The increased fat deposition in Pax7^Δ^ muscle after long-term injury resulted in the need to cut thicker tissue (~30 μm) sections, which also resulted in what appeared to be increased non-specific oil red O (Figure 5A) and BODIPY staining (data not shown). To overcome this issue, we immunostained for perilipin, which is selectively localized to the periphery of lipid droplets and thus specifically marks adipose accumulation. Perilipin staining also revealed a significant increase in adipogenesis in Pax7^Δ^ TA muscle compared to that of Pax7^WT^ mice at 7 and 14 days post-HLI (Figures 5A,B), and this difference persisted out to day 30 post-HLI (Figure 5C). These findings demonstrate that the lack of Pax7^+^ MPCs results in aberrant lipid accumulation, which may contribute to the pathogenesis of PAD.

### 3.6. Fibro/adipogenic progenitors are significantly increased in Pax7^Δ^ mice after ischemia

**Figure 6:**
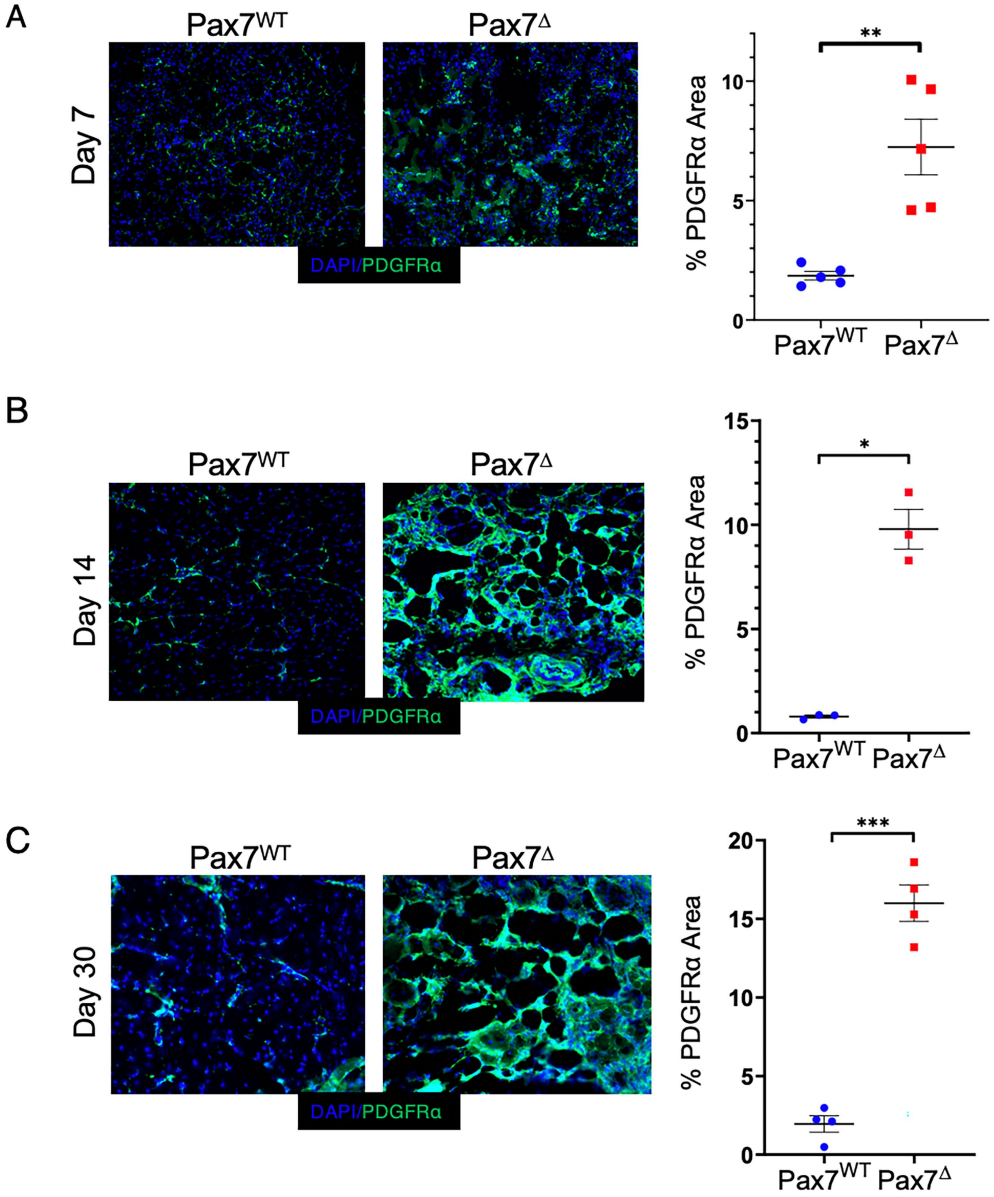
Ablation of Pax7^+^ MPCs in mice results in a significant increase in FAPs in skeletal muscle following ischemia. **(A-C)** PDGFRα staining of the ischemic TA muscle demonstrated significantly increased FAP staining in Pax7^Δ^ mice compared to Pax7^WT^ 7 days (A), 14 days **(B)**, and 30 days **(C)** after HLI surgery (*n* = 3–5 per group). Scale bars = 1 mm. All data shown are means +/-SEM. *p < 0.05; **p < 0.01; ***p < 0.001 by 2-sided *t*-test.

To begin to elucidate the origins of the adipogenic changes observed after ischemia in Pax7^Δ^ mice, we explored the potential contribution of fibro/adipogenic progenitor cells (FAPs) to the phenotype. FAPs have been shown to induce adipogenic changes in skeletal muscle in limb girdle muscular dystrophy type II (33) and in other pathological conditions (22). Additionally, FAPs have been shown to drive adipogenic changes in a variety of metabolic and cardiovascular disorders (26, 27, 34). Staining ischemic muscle from Pax7^Δ^ and Pax7^WT^ mice for the FAP marker PDGFRα (35–37) demonstrated a significant increase in FAPs in Pax7^Δ^ mice that progressively increased over time after ischemia (Figures 6A–C), consistent with the observed temporal increase in adipogenesis (Figure 5). In contrast, PDGFRα^+^ area was unchanged in Pax7^WT^ muscle at all timepoints after ischemia. These findings suggest that increased ischemic skeletal muscle adipogenesis following MPC ablation is driven by FAPs.

### 3.7. Batimastat, an FAP inhibitor, limits adipogenesis and promotes fibrosis after ischemia in the absence of satellite cells

**Figure 7:**
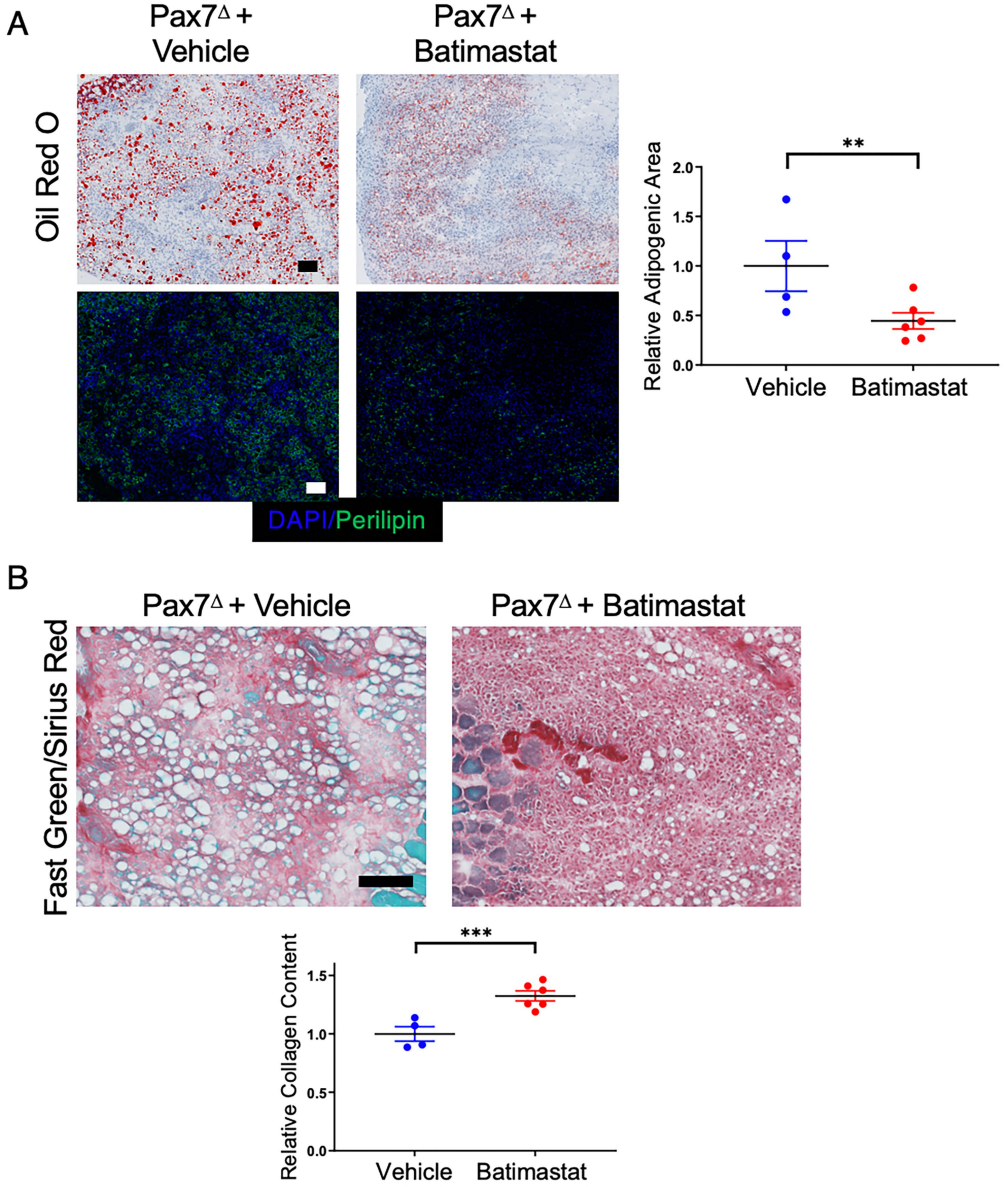
Inhibiting FAPs with batimastat reduces adipogenesis and increases fibrosis after HLI in the absence of satellite cells. **(A)** Batimastat treatment significantly decreased total fat in Pax7^Δ^ ischemic TA muscle as determined by oil red O and perilipin staining 7 days after HLI surgery. **(B)** Fast Green/Sirius Red staining demonstrated a corresponding significant increase in collagen content in Pax7^Δ^ ischemic TA muscle, consistent with a switch from adipogenesis to fibrosis after inhibition of FAPs (*n* = 4–6 per group). Scale bars = 100 μm. All data shown are normalized means +/− SEM. **p < 0.01; ***p < 0.001 by 2-sided *t*-test.

Batimastat is a non-specific MMP inhibitor that has been shown to prevent adipogenesis both in isolated FAP cells *in vitro* and in skeletal muscle *in vivo* (22, 33). We reasoned that if FAPs contribute to adipogenesis after HLI in the absence of satellite cells, then treating ischemic mice with batimastat should limit the amount of lipid deposition. Indeed, treatment of Pax7^Δ^ mice with batimastat during recovery from HLI resulted in a significant decrease in oil red O^+^ and perilipin^+^ area compared to that observed in vehicle-treated Pax7^Δ^ mice (Figure 7A). Notably, this change was accompanied by a corresponding increase in fibrosis (Figure 7B). Despite this clear difference in phenotype, batimastat did not alter the number of FAPs, as indicated by the lack of a difference in PDGFRα staining (Supplementary Figure S3), consistent with previous reports (33). Collectively, these findings suggest that in the absence of satellite cells, ischemia drives FAPs to promote adipogenesis, which may play an important role in the pathophysiology of PAD.

### 3.8. Isolation and differentiation of myoblasts following satellite cell ablation *in vivo* results in defective myogenesis and increased adipogenesis *in vitro*

**Figure 8:**
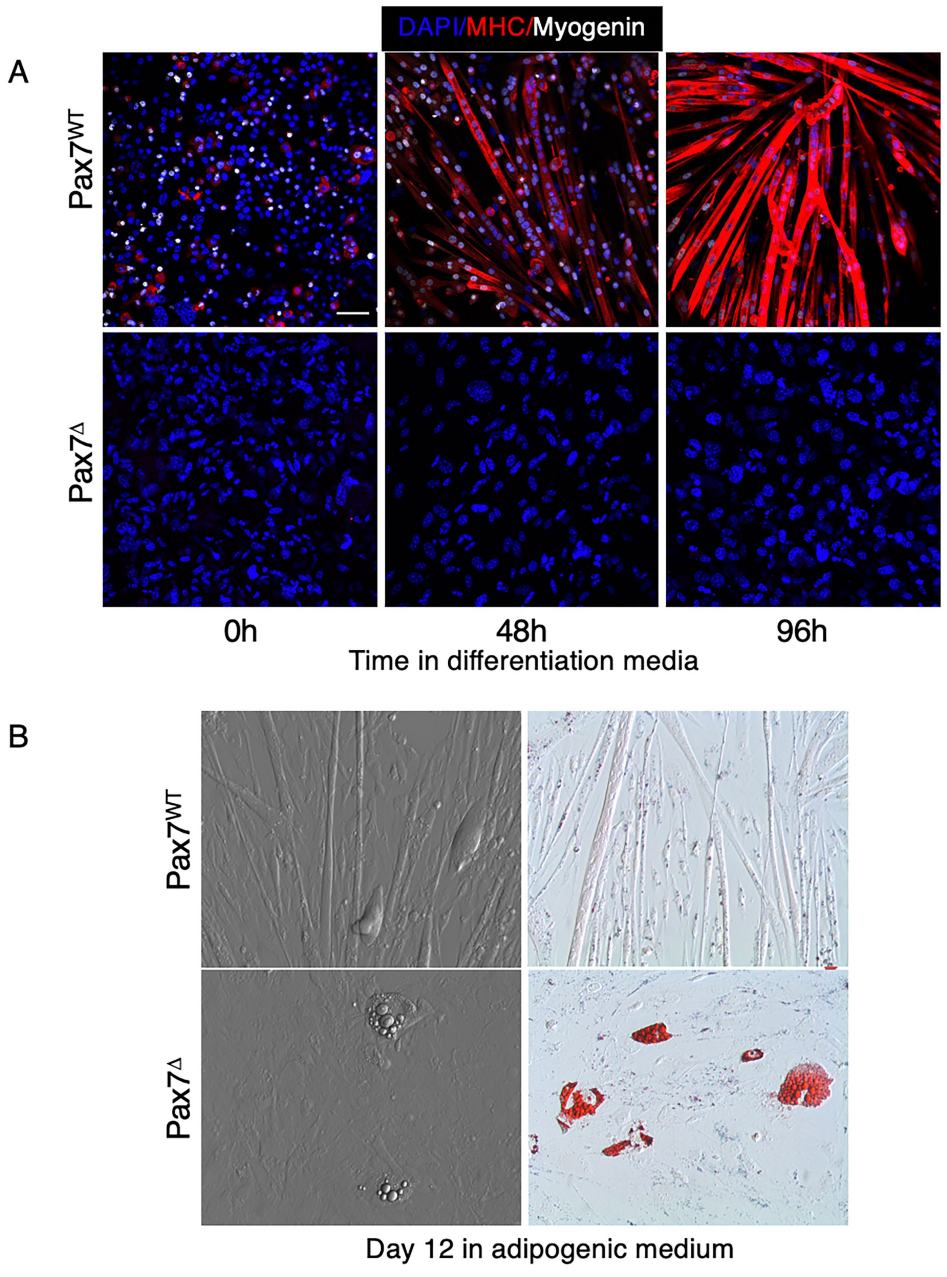
Myoblasts isolated from Pax7-depleted muscle fail to differentiate under hypoxic conditions and display increased adipogenesis. **(A)** Pax7^WT^ myoblasts in differentiation medium expressed myosin heavy chain (MHC) under hypoxia whereas Pax7^Δ^ cells failed to fuse and did not express the early differentiation marker myogenin or MHC. **(B)** When grown in adipogenic medium, Pax7^Δ^ myoblasts had a higher propensity to form oil red O^+^ lipid droplets. Similar results were observed in three independent experiments. Scale bars = 100 μm.

It is well known that myoblasts isolated from whole muscle tissue retain their ability to differentiate and fuse into mature skeletal myotubes *in vitro*. Pax7^+^ satellite cells comprise a small percentage (<10%) of the mononuclear cells isolated from muscle tissue that have the potential to differentiate into muscle (i.e., MPCs). Once MPCs are isolated and plated *in vitro*, satellite cells rapidly lose expression of Pax7 and differentiate into MyoD-expressing committed myoblasts (38). Prior studies have demonstrated that deletion of Pax7^+^ satellite cells *in vitro*, after plating, does not impair myoblast differentiation (39). To our knowledge, however, no studies have examined the effect of *in vivo* ablation of Pax7^+^ cells on subsequent myoblast differentiation *in vitro* and whether this might influence isolated myoblasts to differentiate toward an adipogenic lineage. To test this, mice were treated with either tamoxifen or corn oil for 5 days to ablate Pax7^+^ cells *in vivo*, then muscle was harvested and mononuclear cells/myoblasts were isolated and plated *in vitro*. When cultured in muscle differentiation medium, only cells from Pax7^WT^ mice were able to form mature myotubes, as evidenced by expression of the myogenic regulatory factor myogenin and myosin heavy chain (MHC) (Figure 8A). To determine whether MPCs isolated from Pax7^WT^ or Pax7^Δ^ mice have an increased propensity to differentiate into adipocytes, cells were plated in adipogenic medium. Because increased adipogenesis in Pax7^Δ^ mice was observed *in vivo* in the setting of ischemia, cells were incubated for 12 days under hypoxic conditions to simulate ischemia. Compared to cells from Pax7^WT^ mice, cells isolated from Pax7^Δ^ mice had an increased propensity to form adipocytes, as demonstrated by oil red O staining (Figure 8B). These findings suggest that in the absence of Pax7^+^ cells, Pax7^−^ cells with the potential to fuse and differentiate into muscle are driven toward an adipocyte lineage, although it is unclear whether these cells are FAPs or if they are derived from some other progenitor cell population.

### 3.9. Critical limb ischemia patients have increased adipogenesis and fewer satellite cells in regions of greater ischemia

**Figure 9:**
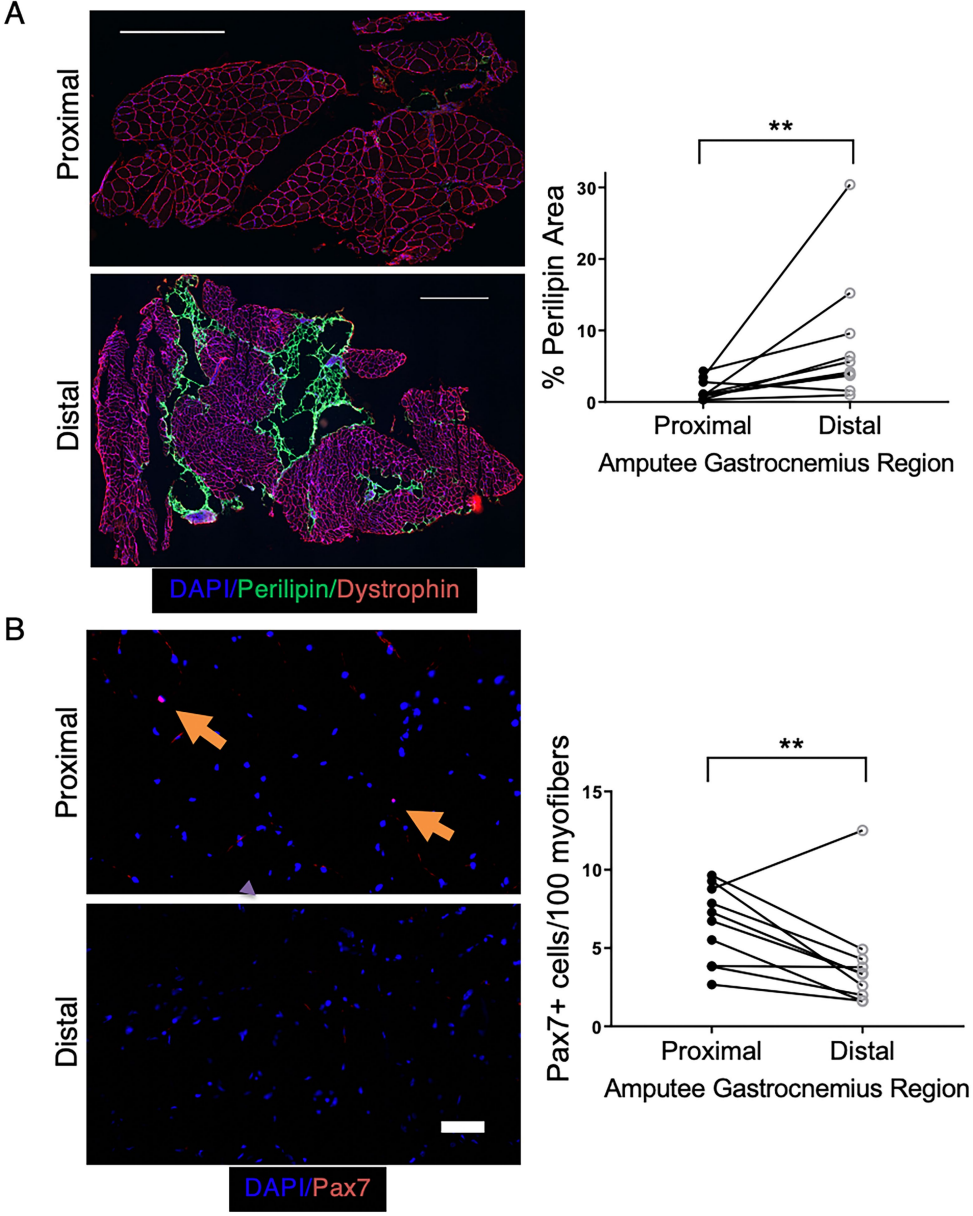
CLTI patients have increased adipogenesis in more ischemic muscle regions that correspond with decreased Pax7^+^ cell numbers. **(A)** Perilipin staining in the gastrocnemius muscle of CLTI patients (*n* = 10) revealed significantly greater fat deposition in more distal ischemic regions. Scale bar = 1 mm **(B)** More ischemic distal regions of the same patients in panel (A) had significantly fewer Pax7^+^ cells. Scale bar = 100 μm. All data shown are paired values from the same patient. **p < 0.01 by a 2-sided ratio paired *t*-test.

To examine whether the adipogenic changes observed in our preclinical model are also seen clinically, we obtained skeletal muscle tissue from CLTI patients undergoing limb amputation. In this setting, tissue that is farthest from the amputation site (distal) is typically the most ischemic, whereas proximal tissue, closer to the amputation site is less ischemic and often relatively healthy. Paired proximal and distal gastrocnemius muscle samples were obtained from 10 CLTI patients undergoing amputation, and adipose area was determined by perilipin staining. Distal, more ischemic muscle displayed significantly greater adipose area (Figure 9A). Because the increase in adipogenic area in our preclinical model was caused by the ablation of satellite cells prior to ischemia, we investigated whether the increased adipogenesis in the regions of greater ischemia corresponded with a loss or reduction in the number of Pax7^+^ cells. Immunostaining for Pax7 was performed on paired proximal and distal skeletal muscle sections from each subject. Although Pax7^+^ cells were still present in all subjects’ distal muscle, we observed significantly fewer Pax7^+^ cells in distal vs. proximal tissue (Figure 9B). These findings support the possibility that chronic limb ischemia results in loss of satellite cell number and/or satellite cell dysfunction, which leads to increased skeletal muscle adipogenesis and may contribute to the pathogenesis of PAD in general and CLTI in particular.

## 4. Discussion

Although surgical and endovascular approaches to revascularization represent the primary strategy to treat PAD, outcomes remain poor, particularly in CLTI, which results in high rates of subsequent amputation (40, 41). Moreover, while experimental pro-angiogenic approaches to improve limb perfusion have shown great promise in preclinical models of hindlimb ischemia, they have proven suboptimal in clinical experience (42, 43). We hypothesized that these poor outcomes might be explained, at least in part, by non-vascular etiologies of CLTI. Our prior results supported this hypothesis by demonstrating that skeletal muscle cell responses to ischemia are independent of blood supply and are strongly influenced by genetic background. However, the role of skeletal muscle regeneration in the response to ischemia and, in particular, the role of muscle progenitor cells in this process, remained unknown. Here, we have demonstrated an absolute requirement for Pax7^+^ skeletal muscle satellite cells in muscle regeneration following ischemic injury. Furthermore, by continuously feeding mice a tamoxifen-containing diet over 30 days post-HLI, we ensured that there was no repopulation of the satellite cell pool (39), and we demonstrated that the regenerative response to ischemia was entirely muscle-dependent. Although one prior study raised the possibility that, following a critical juvenile period, satellite cells were dispensable for regeneration in the postnatal phase, our results are consistent with studies that demonstrate an absolute requirement for satellite cells during regeneration (44, 45), in our case following ischemia-induced muscle injury. Our data demonstrate that complete recovery from ischemia follows a similar time course as skeletal muscle injuries that are cytotoxic and cryogenic in nature (21, 23).

Staining for endothelial cells in mice lacking satellite cells verified that vascular cells were not targeted non-specifically by DTA after tamoxifen treatment and, therefore, that the observed injury was not likely due to loss of vascular supply. Somewhat surprisingly, we found that capillary density was in fact increased in Pax7^Δ^ mice. Although the mechanisms responsible for this effect are not clear, it is possible that capillary proliferation occurred as a compensatory response to the increased tissue destruction (46). One caveat in interpreting this result is that decreased muscle area due to atrophy could have falsely increased apparent vascular density. Future studies will be necessary to fully elucidate the nature of the endothelial response during this process, including examination of endothelial cell proliferation, angiogenesis, and collateralization, which are known to occur in the setting of hindlimb ischemia (47).

Using several complementary approaches (oil red O, BODIPY, perilipin), we demonstrated the novel and important finding that in the absence of Pax7^+^ satellite cells, ischemia induces marked lipid deposition within skeletal muscle. This observation distinguishes the injury in this model from that seen in murine models of muscular dystrophy and cardiotoxin injury, which lack similar adipogenesis. Although the mdx mouse model lacks the extreme fat deposition that is observed in DMD patients (29), a “humanized” mdx model with shortened telomeres and mitochondrial defects did show greater adipogenic changes (48). These lipid deposits are presumed to be pathogenic, because many skeletal muscle diseases are characterized by increased adipogenesis (49). Notably, the adipose deposition observed after complete loss of satellite cells in Pax7^Δ^ mice recapitulated findings seen in muscle tissue samples of CLTI patients, who displayed increased adipogenesis in more ischemic, distal regions of the amputated limb. The mice used in this study were on a C57BL/6 background, a strain in which the skeletal muscle is known to be relatively resistant to ischemic injury (10). Strikingly, the absence of satellite cells completely abrogated the protective effect conferred by C57BL/6 genetic factors, suggesting that satellite cell loss or dysfunction contributes to the CLTI phenotype. Consistent with this observation, we found that more ischemic distal regions of CLTI muscle had significantly fewer Pax7^+^ satellite cells. It is important to note that the mouse phenotype was induced by the complete ablation of satellite cells after tamoxifen treatment, although it is unclear whether partial loss of Pax7^+^ cells would result in a similar phenotype. Although satellite cells were still present in more ischemic regions of CLTI tissue, it is possible that they were dysfunctional and unable to contribute to regeneration. Satellite cell dysfunction may not manifest as a decrease in absolute number, but there may instead be epigenetic, post-transcriptional, and/or post-translational alterations that affect satellite cells’ ability to effectively promote regeneration in CLTI patients. Alternatively, the reduction in Pax7^+^ cell number with ischemia in CLTI may result from a loss due to satellite cell exhaustion reminiscent of phenotypes seen in DMD patients. Future experiments will be necessary to elucidate the exact role that satellite cells play in the pathogenesis of PAD. Gene expression profiling of satellite cells in PAD patients with claudication or CLTI may identify a specific genetic signature that defines the pathophysiology of satellite cells in these conditions. The observed correlation between preclinical and clinical adipose deposition in the setting of limb ischemia supports the biological and clinical relevance of these findings.

FAPs have been shown to play a role in obesity-associated skeletal muscle dysfunction as well as in denervated skeletal muscle (34, 50). We hypothesized that FAPs were responsible for the increased adipogenesis after ischemia in Pax7^Δ^ mice. To explore this possibility, we treated ischemic Pax7^Δ^ mice with batimastat, a small molecule inhibitor of fibroblast activation protein, a dual specificity serine protease. Batimastat has been shown to inhibit adipogenesis resulting from FAP cell differentiation into adipocytes in both isolated FAPs in culture and skeletal muscle *in vivo* in a model of limb girdle muscular dystrophy (33). Indeed, we observed a decrease in the degree of adiposity after batimastat treatment, and this was accompanied by a corresponding increase in fibrosis, supporting the possibility that FAP differentiation into adipocytes was responsible for the observed ischemic lipid deposition. Future studies, such as lineage tracing using an FAP marker like PDGFRα (25), will be necessary to conclusively determine whether FAPs or other progenitor cell types contribute to this fat infiltration. Definitively establishing that FAPs are responsible for the increased skeletal muscle adiposity in the setting of ischemia would likely require a genetic approach, such as ablation of PDGFRα^+^ FAPs. However, ablation of both Pax7^+^ cells and PDGFRα^+^ cells would likely have complex effects that may be difficult to interpret.

Several important questions arise regarding the mechanisms responsible for both the adipogenesis and the switch to a fibrotic phenotype after batimastat treatment. First, what are the paracrine signaling pathways between satellite cells and other muscle progenitor cells, including FAPs, that drive normal myogenesis? Pax7^+^ cells account for a small percentage of total cells in muscle tissue, yet in typical muscle cell isolates, a number of mononuclear cell types have the capacity to fuse and differentiate into myotubes *in vitro*, suggesting that the presence of satellite cells confers on other MPCs (e.g., myoblasts, pericytes, FAPs) the ability to differentiate into functional muscle. This likely involves paracrine signaling mechanisms that remain to be fully elucidated, although PDGF-BB and DLL4 have been implicated in driving pericytes toward a myogenic lineage (51). Second, what are the mechanisms that drive the increased adipogenesis in the absence of satellite cells? Does a suppressive signal from satellite cells to FAPs normally prevent adipogenesis, or does the absence of satellite cells activate another pathway to drive adipogenesis? Third, and equally important, does ischemia contribute to these processes, since adipogenesis does not occur in the non-ischemic limb, or are these pathways driven by aberrant regeneration? Future studies will be necessary to elucidate these mechanisms, and it is hoped that such information would lead to the eventual development of therapies for diseases of aberrant muscle stem cell number and/or function, such as CLTI and DMD. Batimastat provides a potential starting point for development of drugs to inhibit adipogenic changes in skeletal muscle. Although an increase in fibrosis in CLTI in place of adipose tissue may not translate into optimal clinical outcomes, it provides an initial strategy to redirect aberrant MPC differentiation and possibly prevent pathological adipogenesis.

## Data availability statement

The raw data supporting the conclusions of this article will be made available by the authors, without undue reservation.

## Ethics statement

The studies involving human participants were reviewed and approved by Duke University Institutional Review Board. The patients/participants provided their written informed consent to participate in this study. The animal study was reviewed and approved by Duke University Institutional Animal Care and Use Committee.

## Author contributions

HA and CK designed the research study. HA conducted all *in vivo* and *in vitro* experiments and performed data analysis. LO, BG, and KS isolated human skeletal muscle and assisted in human muscle staining and experiments. MP performed animal husbandry, genotyping and HLI surgeries. TM performed histological data analysis and assisted with editing the manuscript. CS and JM conducted muscle force generation experiments. HA wrote the manuscript, and CK co-wrote and edited the manuscript. All authors contributed to the article and approved the submitted version.

## Funding

This study was supported in part by NIH grants HL124444, HL118661, and HL156009 to CK, HL125695 to JM, and by a grant from the Duke University School of Medicine to CK for microCT studies through the Shared Materials Instrumentation Facility. BG was supported by grant F32 HL136125 from the NIH. KS was supported in part by a KL2 award through the Duke Clinical and Translational Science Award TR002553 from the NIH. LO was the recipient of a Eugene A. Stead Student Research Scholarship and a Poindexter Scholars in Basic Sciences Award from the Duke University School of Medicine.

## Conflict of interest

The authors declare that the research was conducted in the absence of any commercial or financial relationships that could be construed as a potential conflict of interest.

## Publisher’s note

All claims expressed in this article are solely those of the authors and do not necessarily represent those of their affiliated organizations, or those of the publisher, the editors and the reviewers. Any product that may be evaluated in this article, or claim that may be made by its manufacturer, is not guaranteed or endorsed by the publisher.
