# Supplementary material for: Muscle progenitor cells are required for skeletal muscle regeneration and prevention of adipogenesis after limb ischemia
Source: Front Cardiovasc Med. 2023 Mar 2;10:1118738. doi: 10.3389/fcvm.2023.1118738 (PMC10017542; doi:10.3389/fcvm.2023.1118738)
Supplement: Supplementary file 1 [file Data_Sheet_1.PDF]

Supplementary Material

Muscle progenitor cells are required for skeletal muscle regeneration and prevention of adipogenesis after limb ischemia

Hasan Abbas, Lindsey A. Olivere, Michael E. Padgett, Cameron A. Schmidt, Brian F. Gilmore, Timothy J. McCord, Kevin W. Southerland, Joseph M. McClung, Christopher D. Kontos*

*** Correspondence:** Corresponding Author: cdkontos@duke.edu

Supplementary Figures

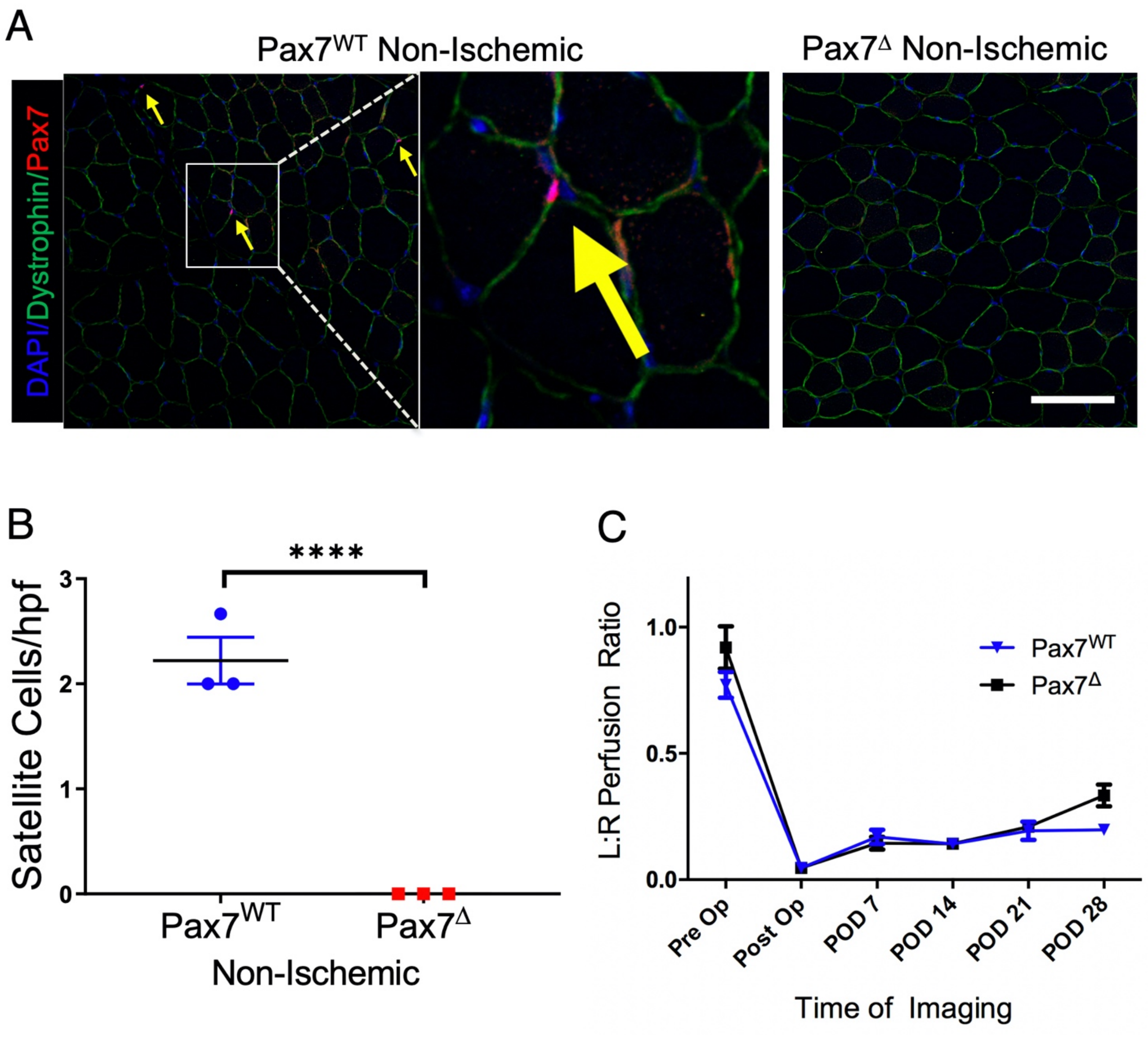

Supplementary Figure S1. Pax7-Cre^{ERT2};ROSA26^{DTA} mice display a complete loss of satellite cells in resting muscle after tamoxifen treatment but no difference in muscle perfusion. (A) Pax7 immunostaining of the non-ischemic TA muscle revealed typical satellite cells at the periphery of resting skeletal muscle (arrows), directly beneath the basal lamina. No Pax7 staining was observed in tamoxifen-treated mice. Scale bar=100 µm. (B) Quantification of satellite cell numbers demonstrated a complete absence of satellite cells following tamoxifen treatment (n=3 per group). ****, $p<0.0001$ by 2-sided *t*-test. (C) Hind limb perfusion, measured as a ratio of perfusion in the ischemic left leg to that in the non-ischemic right leg, was determined at the indicated post-operative days (POD) (n=4 per group). All data shown are normalized means +/- SEM.

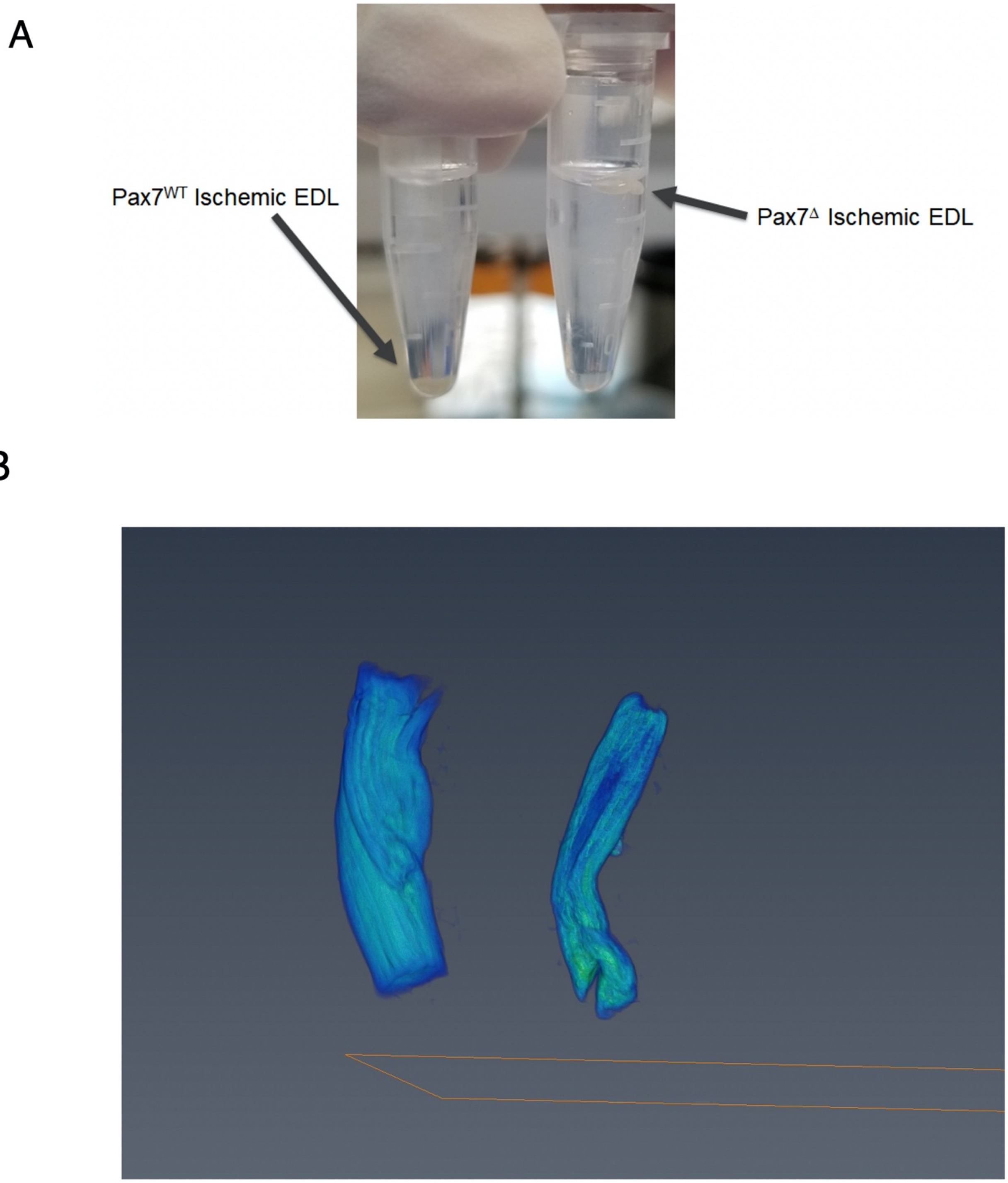

Supplementary Figure S2. Tissue density is reduced in Pax7$^{\Delta}$ EDL muscle 30 days after HLI due to adipogenic changes. (A) Thirty days after HLI, Pax7^{WT} ischemic EDL muscle sinks in aqueous fixative, while the ischemic Pax7$^{\Delta}$ muscle floats, suggesting a reduced soft tissue density due to fat accumulation in the Pax7$^{\Delta}$ muscle. (B) diceCT of 30-day-ischemic Pax7^{WT} EDL muscle (left) showed individual, regenerated, fused muscle fibers while ischemic Pax7$^{\Delta}$ muscle had significant soft tissue fatty changes 30 days after ischemia (green) as well as general muscle atrophy.

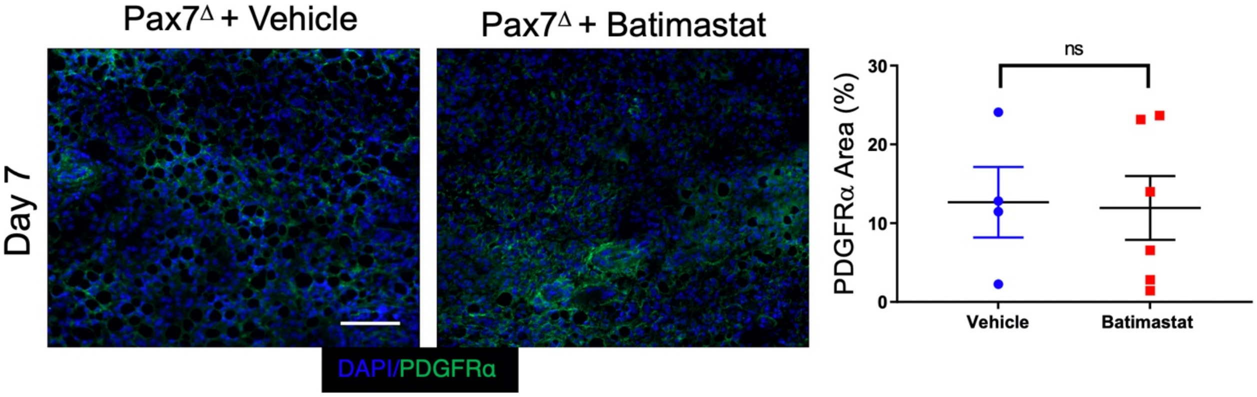

Supplementary Figure S3. Batimastat treatment does not affect infiltration of FAPs. Seven days after HLI, ischemic TA muscle from Pax7$^{\Delta}$ mice treated with vehicle or batimastat was immunostained for PDGFRα (n=4-6/group). Quantification showed no significant difference in PDGFRα^{+} area. Scale bar=100 µm. All data shown are normalized means +/- SEM. ns, not significant (p>0.05) by 2-sided *t*-test.
